# CCN: core regulatory proteins in the microenvironment that affect the metastasis of hepatocellular carcinoma?

**DOI:** 10.18632/oncotarget.6209

**Published:** 2015-10-21

**Authors:** Qingan Jia, Qiongzhu Dong, Lunxiu Qin

**Affiliations:** ^1^ Cancer Center, Institutes of Biomedical Sciences, Fudan University, Shanghai, China; ^2^ Department of General Surgery, Huashan Hospital, Fudan University; Cancer Metastasis Institute, Fudan University, Shanghai, China

**Keywords:** hepatocellular carcinoma, CCN family proteins, metastasis, inflammatory microenvironment

## Abstract

Hepatocellular carcinoma (HCC) results from an underlying chronic liver inflammatory disease, such as chronic hepatitis B or C virus infections, and the general prognosis of patients with HCC still remains extremely dismal because of the high frequency of HCC metastases. Throughout the process of tumor metastasis, tumor cells constantly communicate with the surrounding microenvironment and improve their malignant phenotype. Therefore, there is a strong rationale for targeting the tumor microenvironment as primary treatment of HCC therapies. Recently, CCN family proteins have emerged as localized multitasking signal integrators in the inflammatory microenvironment. In this review, we summarize the current knowledge of CCN family proteins in inflammation and the tumor. We also propose that the CCN family proteins may play a central role in signaling the tumor microenvironment and regulating the metastasis of HCC.

## INTRODUCTION

Liver cancer (mainly hepatocellular carcinoma [HCC]) is a global health problem. In men, HCC is the fifth most frequently diagnosed cancer and the second most frequent cause of cancer death, with more than 80% of HCCs occurring in developing countries [1]. Approximately three-quarters of HCCs are caused by chronic hepatitis B virus (HBV) and hepatitis C virus (HCV) infections [2]. The natural survival time of patients with advanced HCC is only approximately 3 months. Even though many advanced strategy are used in patients with HCC (i.e., early detection, surgical resection, radiofrequency, and liver transplantation), the general prognosis of patients with HCC still remains extremely dismal, and this unfavorable outcome is attributed to the high frequency of HCC metastasis [3].

A growing body of evidence suggests that metastasis is an important event in the progression of many carcinomas and is responsible for as much as 90% of cancer-associated mortalities; however, it remains the most poorly understood component of cancer pathogenesis [4]. The process of metastasis is often viewed as a complex developmental process that can be simplified into two major phases. (1) Early cancerous cells are confined to the primary tumor microenvironment by the continued expression of adhesion molecules and the intact basal lamina. As the cancer progresses, some cells in the primary tumor microenvironment start the process of morphogenesis, termed EMT (Epithelial to Mesenchymal Transition). During the EMT process, the cells acquire mesenchymal cell properties, which facilitate local invasion and metastatic dissemination [5]. (2) While the tumor cells migrate to new target organs, they change back to epithelial cell phenotype in certain premetastastatic niche, termed MET (Mesenchymal to Epithelial Transition), and develop into a metastatic lesion at that distant site [6]. Phenotypic alterations are considered a hallmark for the progression of cancer and provide a new basis for understanding how malignancies develop.

In addition to the common intrinsic heterogeneity of cancer cells that results from genomic instability, a number of phenotypic alterations are induced by the tumor's inflammatory microenvironment [7, 8]. While converting to and from the EMT and MET states in the process of tumor metastasis, tumor cells constantly communicate with the surrounding microenvironment. Therefore, the development of HCC is considered to be the result of different environmental risk factors, each involving different genetic, epigenetic, and chromosomal alterations [9]. The inflammatory condition is now recognized as a hallmark feature of tumor development and metastasis [10]. In contrast to the majority of cancer types that have a relatively simple tumor microenvironment (e.g., breast, lung, and melanoma), HCC is an extraordinarily unique type of cancer in which the inflammatory conditions are present before malignant changes occur. HCC commonly develops after chronic inflammation caused by a HCV infection, HBV infection, certain metabolic liver diseases, or alcoholic injury, all of which create a tumor-permissive milieu. The tumor microenvironment of HCC is not only composed of cells typically seen in other cancers (i.e., tumor cells, fibroblasts, invading inflammatory/immune cells, reactive oxygen and nitrogen species, tumor-associated cytokines, and chemokines). The microenvironment of HCC is also composed of hepatitis virus-associated inflammatory cytokines, chemokines, and growth factors, all of which create a dynamic microenvironment that contributes to the development of HCC [11]. Therefore, the liver tumor microenvironment is much more complex than that of any other cancer. The bidirectional cross talk between the tumor cells and the tumor microenvironment plays a fundamental role in the progression of the tumor and, concomitantly, the survival of patients [12, 13, 14].

The goal of this review is to highlight the role of the microenvironment's inflammatory immune responses in HCC metastases and to point out the prospective areas for future research and possible precision medicine approaches to control HCC metastasis. Recently, most studies have established that CCN family proteins are localized multitasking signal integrators in the inflammatory microenvironment [15]. In this review, we summarize the findings on CCN family proteins in inflammation and in the tumor. We also propose that the CCN family proteins play a central role in regulating the tumor microenvironment and metastasis of HCC.

## CCN FAMILY PROTEINS

**Figure 1:**
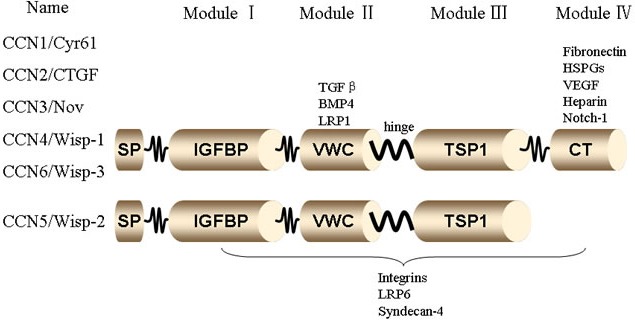
Structure of CCN family members The CCN family members, CCN1 (Cyr61), CCN2 (CTGF), CCN3 (NOV), CCN4 (WISP-1), CCN5 (WISP-2), and CCN6 (WISP-3) have a shared structure, consisting of a secretory signal peptide (SP), an IGF binding domain (IGFBP), a von Willebrand type C domain (VWC), a thrombospondin-1 domain (TSP1), and a cystine knot domain (CT). The domains are linked by hinge regions and are susceptible to protease cleavage.

The CCN family, first described by P. Bork in 1993, is a small, six-member family of cysteine-rich regulatory proteins found in humans. The first three members described: CCN1 (Cysteine rich 61, CYR61), CCN2 (Connective Tissue Growth Factor, CTGF), CCN3 (Nephroblastoma Overexpressed, NOV), provided the acronym for the CCN family. CCN4 (Wnt1-Inducible Signaling Pathway Proteins, WISP-1), CCN5 (WISP-2), and CCN6 (WISP-3) were subsequently identified [16]. Together, the six members form the small group of structurally similar matrix proteins of approximately 40 kDa that regulate numerous biological phenomena [17; 18]. Members of this secreted protein family share a multimodular structure with an N-terminal secretory signal domain followed by four conserved domains with homologies to Module I (insulin-like growth factor binding proteins, IGFBP), Module II (von Willebrand factor type C repeat, VWC), Module III (thrombospondin type I repeat, TSP), and Module IV (carboxy-terminal domain, CT) containing a cystine knot [19] (Figure 1). Because the CCN proteins are characterized by four unique globular modules that share homology with functional domains of various extracellular proteins, this family does not behave like traditional growth factors or cytokines. The proteins do not appear to have a unique receptor for high-affinity binding and signal transduction. In recent years, there has been much interest in this family of proteins, mainly to understand the function of their multimodular structures [20].

It may be more accurate to think of CCN family proteins as matricellular proteins that modulate cell-matrix interaction to modify the cellular phenotype [21]. CCN family proteins** are not ubiquitously or constitutively expressed, but are under strict regulation by a number of extracellular stimuli. Many studies have shown how physiological and pathological events acting on each catalytic domain of CCNs affect differentiation [22], adhesion [23, 24], migration [25, 26], mitogenesis [27], chemotaxis [28] and angiogenesis [29]. Furthermore, CCN proteins participate in the development of cartilage and bone, the nervous system, and hematopoietic stem cells [30, 31, 32]. Many of the above-mentioned phenomena are also associated with cancer [33], implying that these proteins may also have a role in malignancy. Chronic liver damage is a paradigm of cancer-related inflammation caused by hepatitis virus infection, alcoholic injury, etc. Chemokines, cytokines, growth factors, and inflammatory mediators regulate the infrastructure of the tumor inflammatory microenvironment by deciding the signal transduction and functional outcome of tumor cells. This review summarizes the relevant literature on CCNs as core regulatory proteins that orchestrate the inflammatory microenvironment.

## CCN FAMILY PROTEINS IN HCC

### Altered expression of CCN proteins in HCC

In HCC, the expression levels of CCN1, CCN2, CCN3, and CCN4 are closely correlated with certain biological characteristics and clinical features, including venous invasion, cellular differentiation, TNM stage, disease-free survival, and overall survival. All of these features are valuable in determining prognosis [34, 35, 36, 37, 38]. Chen et al [39] showed that CCN1 expression was downregulated in human HCC, and CCN1 suppressed hepatocarcinogenesis by inhibiting EGFR-dependent hepatocyte compensatory proliferation. Paradoxically, Zeng et al [35] reported CCN1 mRNA in carcinoma tissues were significantly higher than those in para-cancerous normal liver tissues, which play an important role in hepatocellular carcinogenesis and correlate with recurrence and metastasis of HCC. Li et al [40] also found that CCN1 was a direct downstream target of Wnt/β-catenin signaling, activation of β-catenin signaling elevated the level of CCN1 in HCC cells, which promoted the progression of HCC. It was proved expression of CCN2 was an independent factor associated with shorter OS in HCC [34, 41, 42]. Hou et al [43] proved CCN2 were related to the formation of HCC BM (Bone Metastases), which could serve as a potential prognostic biomarkers for BM from HCC [44]. While, the inhibition of CCN2 expression can block the progression of HCC [45]. Enhanced expression of CCN3 were found in HCC samples when compared to levels in matched non-cancerous tissues, and these results suggest that Nov was associated with the development of tumors in the live [34, 37]. CCN4 was also proved as a negative correlation with OS in HCC [34], contributing to the Wnt pathway activation [46]. No significant difference in CCN5 was found between HCC samples and matched-pair normal liver samples [34]. Although, it was reported overexpression of the hepatitis C viral core protein in Huh-7 cells caused an upregulation of CCN5 and increased proliferation of the cells [47], it was still hard to tell the role of CCN5 in HCC malignant phenotype. Report of CCN6 expression in HCC is still lacking. Our colleagues Ye et al [48] analyzed the cDNA microarray-based gene expression profile of HCC samples with or without extra-hepatic metastases. They found that CCN3 was significantly upregulated in metastatic HCC. Recently, we confirmed that high expression levels of CCN2 and CCN3 were correlated with poor prognosis in HCC patients using tissue microarray (unpublished data). We also analyzed the profiles of tissue from patients with oxaliplatin-resistant HCC and found significantly upregulated expression of CCN1 and CCN3 [49]. The expression of CCN family proteins in HCC clinical sample was summarized in Table 1.

**Table 1:** Expression of CCN family members in Hepatocellular Carcinoma

CCN Protein	Model	Effect	Ref.
CCN1/Cyr61	Clinical sample	Negatively correlated with HCC development	[34, 39, 115, 116]
CCN2/CTGF	Clinical sample	Positively correlated with HCC development	[35, 40]
CCN3/NOV	Clinical sample	Positively correlated with HCC development	[37, 38, 41, 44]
CCN4/WISP-1	Clinical sample	Positively correlated with HCC development	[34, 37]
CCN5/WISP-2	Clinical sample	Negatively correlated with HCC development	[34]
CCN6/WISP-3	-	No significant differenceLack of data	-

### Altered expression of CCN proteins in HCC stroma

CCN family proteins paracrined by HCC stromal cells are also closely correlated with HCC progression. Our group has collaborated with the United States' National Cancer Institute to compare the gene expression profiles of noncancerous surrounding hepatic tissue from primary HCC with or without metastases. This study found a unique change in the gene expression profile in the noncancerous hepatic tissues of metastatic tumors with more than 30% of them associated with inflammation and/or immune response functions. CCN3 was significantly upregulated compared with the liver tissue of non-metastatic HCC. CCN1, CCN2 also have corresponding upregulation, and CCN4 was down regulated significantly [50]. We confirmed this finding of higher expression levels of CCN2 and CCN3 in the noncancerous surrounding hepatic tissue from primary metastatic HCC using tissue microarrays (unpublished data). Kim [51] et al found that CCN1 is highly accumulated in the hepatocytes of human cirrhotic livers where it acts as a key regulator in the accumulation of reactive oxygen species (ROS) [52]. High levels of ROS could trigger the activation of macrophages and cause immunosuppression in the tumor microenvironment. The involvement of platelets in tumor progression has been well recognized for decades. CCN1 is released from or produced by activated platelets at levels more than 20-fold higher than any other growth factors [53], suggesting that platelets constitute a novel source of CCN1 release and may play central roles in tumor progression [54]. Higher serum CCN2 concentrations in patients with HCC are most likely due to the active fibrogenic tissue matrix surrounding the tumor [55] and played an important role in the progression of HCC and relationship with angiogenesis of HCC [56]. They also found that CCN2 silencing induced a sustained antifibrotic effect in a mouse model [57].

CCN proteins are inducible immediate early genes involved in diverse physiological and pathological processes after exposure to various stimuli. In unpublished data from our lab, we found higher expression of CCN proteins in the parenchyma and stroma of HCC tumors with extra-hepatic metastases. Taken together, this suggests that the malignant potential of HCC may be determined not only by the intrinsic heterogeneity resulting from genomic instability, but also may be determined by the tumor microenvironment, which is influenced by CCN proteins.

## THE RELATIONSHIP BETWEEN INFLAMMATION MICROENVIRONMENT AND CCN PROTEINS

One hypothesis views tumors as non-healing wounds with a close link to the chronic inflammatory microenvironment. Supporting this idea, HCC typically occurs in an inflammatory fibrotic microenvironment in the liver, caused by chronic liver damage [58]. Global gene expression profiling has revealed that the tumor microenvironment is important in the biologic and prognostic classification of the tumor [50]. Inflammation is a complex process regulated by an extraordinarily large variety of immune cells and a cascade of cytokines and chemokines synthesized in the inflammatory region. This is especially true in HCC. Divella [59] et al demonstrated that tumor heterogeneity is closely linked with inflammation in the presence of HBV/HCV and confirmed that the presence of viral infections increases the risk for HCC development. Shin [60] et al found that HCV core proteins could promote hepatic fibrogenesis via upregulation of CCN2. Samuel [61] et al found that hepatitis B viral protein (HBx)-expressing hepatocytes showed an increased expression of CCN2, which is a major cause of liver fibrosis that eventually leads to cirrhosis and HCC. Bian et al [62] found that CCN1 expression in hepatocytes is involved in the hepatic proinflammatory response and macrophage infiltration.

The multimodular structure of the CCN proteins allows them to bind and interact with a broad range of other extracellular proteins, including integrins, heparin sulfate proteoglycans (HSPGs), extracellular matrix proteins (e.g., fibronectin and fibulin 1C), receptors (e.g., Notch1 and tropomyosin receptor kinase A [TrkA]), low-density lipoprotein receptor-related proteins (LRPs), and cytokines (e.g., bone morphogenetic proteins, transforming growth factor-β [TGF-β], connexin 43, and vascular endothelial growth factor). This flexibility enables CCN proteins to play a core role in inflammatory cytokine synthesis and immune cell recruitment during the inflammatory process [63]. (Figure 1)

### Cytokine and chemokine secretion induced by CCN proteins

There is growing evidence that the expression of CCN proteins is regulated by cytokines [64, 65, 66, 67, 68, 69]. Reciprocally, CCN proteins could differentially modulate the secretion of cytokines through a variety of signaling pathways. For example, CCN1 can activate integrin-nuclear factor κB (NFκB) signaling in macrophages, leading to the expression of multiple proinflammatory signals, including upregulation of cytokines (tumor necrosis factor α [TNFα], interleukin [IL]-1α, IL-1β, IL-6, IL-12b), chemokines (MIP-1α, MCP-3, Gro1, Gro2, IP-10), regulators of oxidative stress, and the complement system (inducible nitric oxide synthase, C3) [70, 71]. In gastric cancer cells, CCN1 can upregulate COX-2 and the chemokine receptors CXCR1 and CXCR2 via NF-κB and Scr/PI3K/Akt-dependent pathways, respectively [72, 73]. In other cell systems (for example, stellate cells, cardiomyocytes, and mesangial cells), CCN2 can regulate the proinflammatory cytokines TNFα and IL-6 and the chemokines MCP1 and IL-8 through NF-κB signaling during inflammation [74, 75, 76, 77]. CCN3 specifically induces the expression of the proinflammatory chemokines MCP1 and GROα in primary cultured astrocytes through distinct integrins and signaling mechanisms, such as activation of integrin β1/Rho/ROCK/JNK/NF-κB and integrin β5/Rho/ROCK/p38/NF-κB pathways, respectively [78]. In cancer cells and vascular smooth muscle cells, overexpression of CCN4 and CCN5 leads to downregulation of matrix metalloproteinase (MMP)-1 and MMP-2, respectively [79, 80]. There is clear evidence that CCN family proteins can activate proinflammatory pathways, making them a potentially important signal in cancer cells.

### Cytokine and chemokine activity orchestrated by CCN proteins

According to the published literature, CCN proteins display proinflammatory and anti-inflammatory activities in a cell type-specific manner, indicating that this family of proteins could be a core modulator of the inflammatory microenvironment. CCN1, CCN2, and CCN3 are able to unmask the cytotoxicity of TNFα and lymphotoxin-α, as well as enhance the apoptotic activity of the Fas ligand (FasL) and the TNF-related apoptosis-inducing ligand (TRAIL) in normal human skin fibroblasts [81]. Paradoxically, CCN1 contributes to prostate cancer cell proliferation and TRAIL-induced apoptosis, the latter effect through interaction with integrins avβ3 and a6β4, syndecan-4, and PKCα-dependent signaling [82]. CCN2 can modulate the bioavailability and/or activity of TGF-β through its VWC domain and thereby potentiate TGF-β receptor binding and signaling [83, 84]. In cultured astrocytes, CCN3 added in combination with TNFα synergizes the stimulatory effect of TNFα on GROα expression [63]. CCN4 exerts pro-mitogenic and pro-survival effects, partly by antagonizing TNFα-mediated cardiomyocyte death [85].

### Immune cells orchestrated by CCN proteins

**Figure 2:**
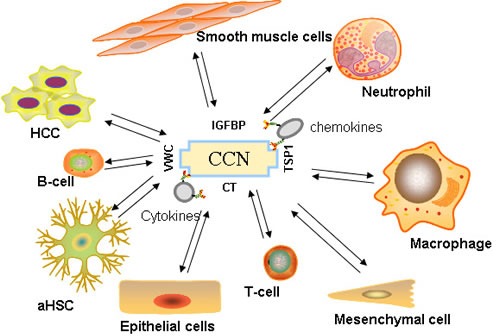
Immune signaling orchestrated by CCN proteins Cytokines, chemokines, stromal cells, and other immune cells in the HCC tumor's inflammatory microenvironment are influenced by CCN proteins.

The most ubiquitous function of the CCN proteins is their ability to support adhesion and regulate migration, proliferation, survival, and apoptosis. All of these factors contribute to the recruitment of immune cells or angiogenesis, which are crucial processes that involve the inflammatory microenvironment. Leukocyte transmigration is a multi-step event that is considered a hallmark of inflammation. CCN1 can induce cell adhesion and thereby contribute to the recruitment of leukocytes and macrophages to the inflammatory sites in an integrin-dependent manner [86]. In vivo, CCN2 also markedly promotes infiltration of T lymphocytes, monocytes, and macrophages into the renal interstitium [74]. Chen [24] et al has shown that prostate cancer-derived CCN3 can induce M2 macrophage infiltration, increase tumor growth, and support tumor-associated angiogenesis in the tumor microenvironment. CCN1 can promote the integrin-dependent recruitment of CD34^+^ progenitor cells to endothelial cells, thereby enhancing endothelial proliferation and neovascularization in vitro and in vivo [87]. CCN4 [88], CCN5 [80], and CCN6 [89] seem to play a preventive role in tumor progression. Antonio et al [90] found that inhibiting CCN2 expression can interrupt the cross talk between HCC and fibroblasts, leading to a significant reduction of HCC growth and dissemination. (Figure 2)

## ROLE OF INFLAMMATORY MICROENVIRONMENT IN THE MALIGNANT PHENOTYPE OF HCC

Uncontrolled or sustained inflammation is the underlying cause of or actively contributes to the progression of tumor [91, 92, 93, 94]. The liver tumor inflammatory microenvironment is a complex mixture of HCC cells within the ECM, combined with a complex mix of stromal cells and the proteins they secrete. Together, these elements contribute to the HCC epigenetic alterations. Cancer cells do not manifest the disease alone and the stroma is inappropriately activated in cancer to contribute to malignant characteristics of tumor cells. The stroma and the tumor cells create a complex system with reciprocal signaling [95]. The condition of HCC inflammatory microenvironment is now recognized as a hallmark feature of tumor development and metastasis [8]. The functions of CCN proteins are involved in the regulations of inflammatory microenvironment [15, 96, 97]. In particular, CCNs expression is regulated by cytokines and immune cells, and that, reciprocally, CCNs may regulate cytokines, chemokines expression and immune cell functions. So, CCN proteins can represent a new class of mediators that contribute to the fine tuning of HCC's inflammatory regulations [45, 98, 99].

## NEW APPROACHES TO TARGET CCN PROTEINS IN HCC

Technological advancements in the molecular characterization of cancers have enabled researchers to identify an increasing number of key molecular drivers of cancer progression [100, 101, 102, 103]. There have been several examples of successful monoclonal antibody and small molecule drug, particularly those targeting key molecular pathways in the field of oncology over the past decade [104; 105; 106]. To date, Sunitinib, Brivanib, Linifanib, Sorafenib and other molecules tested as second-line therapies for advanced HCC, failed to demonstrate an increased survival compared to placebo [107]. What are the possible reasons for the failure? As our understanding of the complex interplay between tumor and stroma, different components of the tumor's inflammatory microenvironment play a significant role in each step of cancer progression, such as angiogenesis, invasion, and metastasis [108]. HCC emerges late in the course of the HBV or HCV infection, after years of inflammation and constant regeneration have led to substantial fibrosis and a constellation of molecular changes that culminate in cancer. The prevailing hypothesis is that HCC is nearly always the result of inflammation; therefore, future therapeutic strategies should focus on targeting this inflammation.

Abnormal levels of CCN proteins have been widely reported in human cancers and stromal cells in the tumor microenvironment where they can either enhance or inhibit cancer cell growth [109]. CCN proteins, along with other proteins such as thrombospondins and osteopontins, are matricellular proteins that modify the signaling pathways of other molecules, especially those associated with inflammation. Because CCN proteins influence multiple physiological and pathological processes, therapy targeted to this protein family has many potential clinical applications for cancer, especially HCC.

FG-3019 is a monoclonal antibody against CCN2 that was tested in locally advanced or metastatic pancreatic cancer in a phase I study*.* The experimental drug was administered combination with Erlotinib and Gemcitabine; no toxicity was found relating to FG-3019. Patients progressed after a median time of 3.7 months and had a median survival of 9.4 months [110]. FG-3019 has been tested previously in a phase I study in patients with microalbuminuric diabetic kidney disease. Here, the researchers demonstrated a saturable pathway for drug elimination, minimal infusion adverse events, and no significant drug-attributable adverse effects over the year of follow-up [111]. Neesse [112] et al found that tumor-stromal interactions critically contribute to innate drug resistance in pancreatic cancer and that targeting tumor microenvironmental factors through CCN2 played an important role in treatment responses. Finger [113] et al offered the first preclinical validation of anti-CCN2 therapy for the treatment of advanced melanoma. Because of its reasonable toxicity spectrum, FG3091 is ready to be tested in phase II trials in patients with pancreatic cancer [114]. These preclinical studies provide strong evidence for targeting CCN proteins in HCC therapy. Inhibition of CCN proteins, especially CCN2, represents a new therapeutic strategy that may be used alone or in combination with current treatments for HCC.

## SUMMARY AND PROSPECTS

HCC is nearly always the result of underlying chronic liver inflammation, usually viral hepatitis. The choice and outcome of treatment depend on the extent of the tumor and the overall condition of the liver. Currently, drug development in HCC remains focusing on HCC itself, which could be ignorance of the importance of the HCC microenvironment driving a tumor-permissive milieu. Throughout the process of tumor metastasis, tumor cells constantly communicate with the surrounding microenvironment and improve their malignant potential. Now that the inflammatory condition is recognized as a hallmark feature of tumor development and metastasis, better therapies will hopefully follow.

CCN family proteins have emerged as localized multitasking signal integrators in the inflammatory microenvironment. CCN proteins are modular proteins containing as many as four distinct functional domains involved in binding to cytokines, chemokines, and cell surfaces molecules, including integrins, HSPGs, and LRPs et al, which play important roles in tumorigenesis, proliferation, adhesion, migration, and survival. Although most CCN family members were discovered more than a decade ago, the precise physiological function and mechanism of action of these proteins remain elusive, especially for the tumor microenvironment. Future directions should be focused on how the inflammatory microenvironment regulates tumor progression, especially in HCC. A thorough understanding of the biological functions of CCN proteins would provide insights into their roles in numerous cellular processes and offer opportunities to develop potential therapeutics.

## Abbreviations

HCC: hepatocellular carcinoma
HBV: hepatitis B virus
HCV: hepatitis C virus
EMT: epithelial to mesenchymal transition
CYR61: cysteine rich 61
CTGF: connective tissue growth factor
NOV: nephroblastoma overexpressed
WISP-1: wnt1-inducible signaling pathway proteins
WISP-2: wnt2-inducible signaling pathway proteins
WISP-3: wnt1-inducible signaling pathway proteins
IGFBP: insulin-like growth factor binding proteins
VWC: von willebrand factor type C repeat
TSP: thrombospondin type I repeat
CT: carboxy-terminal domain
ROS: reactive oxygen species
HBx: hepatitis B viral protein
HSPGs: heparin sulfate proteoglycans
